# Fatal epistaxis in a case of common variable immunodeficiency: A case report and review of the literature

**DOI:** 10.1002/ccr3.5602

**Published:** 2022-03-23

**Authors:** Lucy Barber, Anthony Jordan, Richard Douglas

**Affiliations:** ^1^ 58991 Department of Otorhinolaryngology, Head and Neck Surgery Auckland City Hospital Auckland New Zealand; ^2^ Department of General Medicine Clinical Immunology and Allergy Auckland City Hospital Auckland New Zealand

**Keywords:** carotid artery aneurysm, chronic rhinosinusitis, common variable immunodeficiency, granulomatous inflammation

## Abstract

Common variable immunodeficiency (CVID) is a primary immunodeficiency disease. We present a case of a patient with CVID complicated by rhinosinusitis with granulomatous inflammation. Treatment for this patient was challenging with regards recognition of the granulomatous manifestation as well as treatment in the setting immunodeficiency and was ultimately unsuccessful.

## Graphical abstract

Granulomatous inflammation affecting the sinonasal mucosa should be considered in patients with CVID and recalcitrant chronic rhinosinusitis. Appropriate treatment is challenging, balancing immune suppression with an existing immune defect.

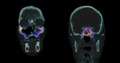

## 1 INTRODUCTION

Common variable immunodeficiency (CVID) is the most common form of severe primary antibody deficiency.^1^, ^2^ It is characterized by reduced serum immunoglobulins and defective antibodies responses, leaving patients susceptible to infections.^1^, ^2^, ^3^, ^4^ A subset of patients also have granulomatous and/or autoimmune manifestations.^3^ The current treatment for CVID is immunoglobulin replacement therapy in the form of intravenous or subcutaneous immunoglobulin.^2^ This form of therapy is not effective in controlling the granulomatous manifestations of this disease.^3^ Granulomatous inflammation affecting the sinonasal mucosa in patients with CVID has not previously been described in the literature, nor has fatal epistaxis secondary to internal carotid artery pseudoaneurysm.

## 2 CASE DESCRIPTION

We present a case of a male patient who was diagnosed with CVID at age 28. He was managed at his regional hospital for many years with four weekly injections of intravenous immunoglobulin (IVIG). After developing chronic rhinosinusitis that was unresponsive to medical therapy, he underwent functional endoscopic sinus surgery (FESS) at age 48. His symptoms significantly improved following this procedure.

Subsequently, his symptoms worsened and a revision FESS and nasal mucosal biopsies were performed. Cultures from the operating room were positive for *Proteus mirabilis*, Coagulase negative *Staphylococcus*, *Corynebacterium* and *Haemophilus influenzae*. There was no evidence of fungal elements suggesting an invasive fungal process. His symptoms failed to improve after the second surgery.

Two weeks following this procedure he presented with fevers and severe pain over his maxillary sinuses. Computed tomography (CT) showed left lateral maxillary wall osteitis and generalized thickening of the sinonasal mucosa. He was commenced on a 6 week course of intravenous Tazocin. After limited improvement, he was then changed to vancomycin and ciprofloxacin for 6 weeks. He returned to theatre for further biopsies of the left maxilla and repair of an oroantral fistula.

**FIGURE 1:**
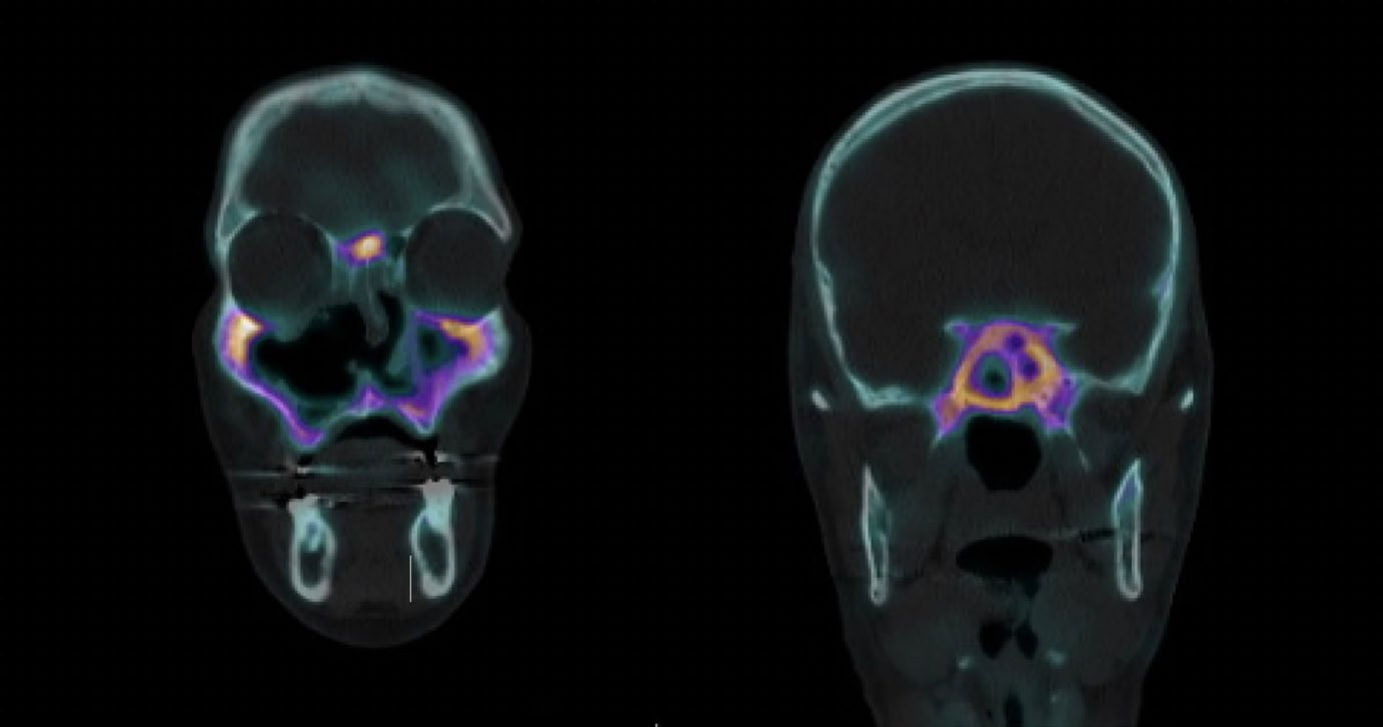
Scintigraphy showed gallium uptake in the maxillary, frontal, and sphenoid sinuses consistent with probable osteomyelitis on the background of known sinusitis

His facial pain failed to settle. Scintigraphy showed contiguous gallium uptake about the maxillary, right frontal and sphenoid sinuses consistent with osteomyelitis (Figure 1). After consultation with an infectious diseases physician, he was started on a trial of 3 month of intravenous ceftriaxone and a concurrent course of oral clindamycin for 3 months.

In December 2018, the patient was referred to the rhinology clinic in our center, and as his facial pain and nasal discharge had not settled, he was booked for further sinus surgery and biopsies. During the surgery, it was noted that there was an extensive amount of necrotic tissue in both maxillary sinuses and mucopurulent discharge was drained from frontal sinuses. Postoperatively, he was seen by the pain team and discharged from hospital within a week of the surgery on several analgesics.

The histology report described inflammation of the nasal epithelium and adjacent stromal tissues with severe foci of granulomatous inflammation along with evidence of arteritis with endothelial proliferation and full thickness inflammation. Serum antineutrophil cytoplasmic antibodies (ANCA) was negative. The clear histological evidence of granulomatous disease changed the treatment focus, and he was started on prednisone and cotrimoxazole, and within a few days he was feeling considerably better.

However, 2 weeks later, he presented acutely with epistaxis, which was very brisk but had stopped spontaneously prior to arrival. Nasoendoscopy showed copious crusting and purulent discharge in the nasal cavity, but no discrete bleeding point was identified on nasal examination under anesthesia.

**FIGURE 2:**
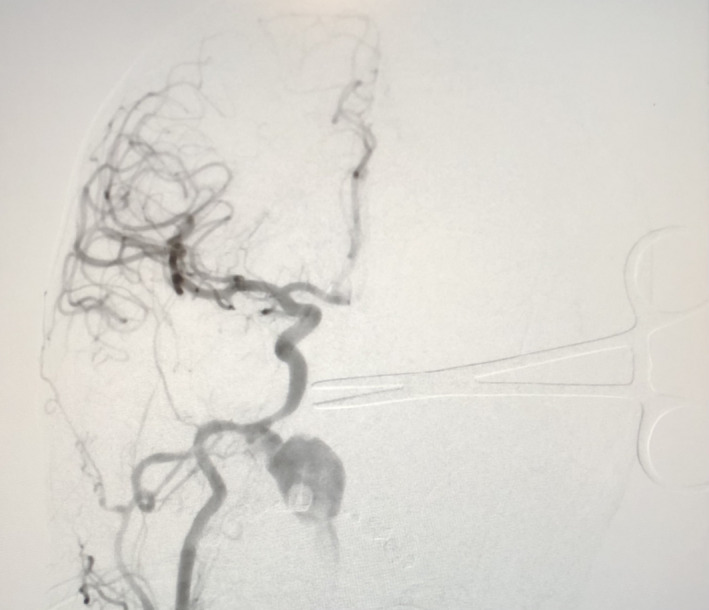
Angiography of the right common carotid angiography demonstrated a large pseudoaneurysm filling from the junction of the right petrous and laceral segments of the internal carotid artery with active bleeding

**FIGURE 3:**
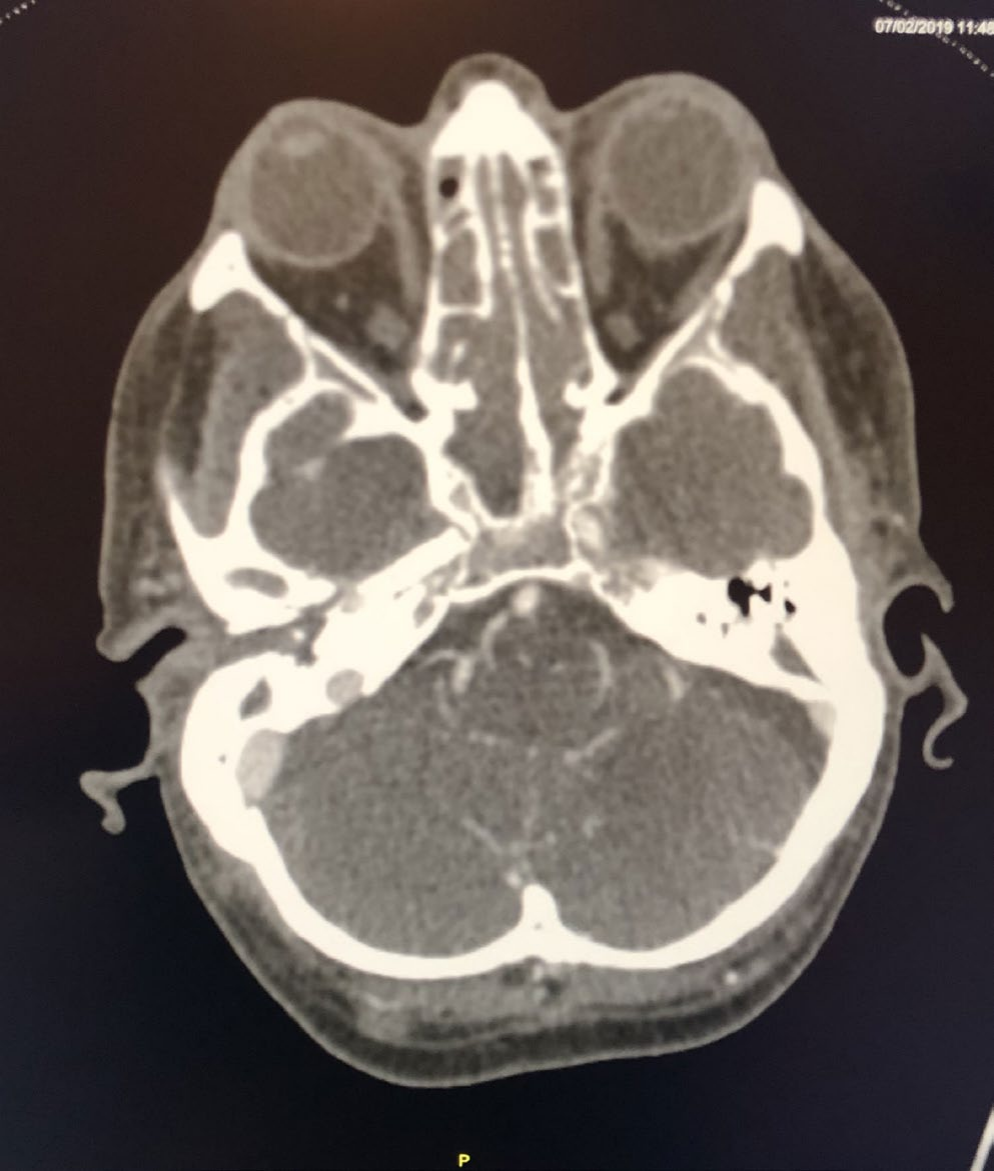
CT angiogram post stent placement

Three days following this acute admission, he was clinically improving when he suddenly developed fulminant epistaxis. Attempts at packing the nose with bilateral Foley catheters and ribbon gauze packing were unsuccessful. He lost consciousness within 2 minutes of the onset of the epistaxis and arrested. He was resuscitated and aggressively transfused. After 40 minutes of cardiopulmonary resuscitation (CPR), spontaneous circulation was achieved. During this time, he was intubated and his oropharynx and nasopharynx were packed which tamponaded the bleeding. Once sufficiently stable, he proceeded to the interventional radiology suite. Angiography of the right common carotid demonstrated a large pseudoaneurysm filling from the junction of the right petrous and laceral segments of the internal carotid artery. Active extravasation into the soft tissues of the nasopharynx was evident (Figure 2). Two stents were placed, and the bleeding was controlled (Figure 3).

He was subsequently admitted to the intensive care unit where further blood products were administered, and he was started on low dose noradrenaline for blood pressure support. He had bilateral infiltrates on chest x‐ray which was consistent with aspiration of blood. CT angiogram revealed no intracranial hemorrhage or definite intracranial ischemic changes and the stent appeared to be patent. However, an EEG showed a significant diffuse disturbance of the background cerebral activity. On de‐sedation, he had myoclonus. The decision was made to extubate him. He died the following morning from hypoxic ischemic encephalopathy secondary to a hypovolemic cardiac arrest.

## 3 DISCUSSION

Common variable immunodeficiency is a primary immunodeficiency which is characterized by low levels of immunoglobulins and failure to make appropriate antibodies following infection or immunization.^1^, ^2^, ^3^, ^4^ Variable T‐cell abnormalities can be present, including cytokine defects and poor lymphocyte proliferation.^3^, ^4^ Most patients present with a history of recurrent infections but they can also present with systemic granulomatosis, autoimmune manifestations, malignancies, and gastrointestinal disease.^3^, ^5^ The diagnosis is typically made between age 20 and 40 years.^6^ The pathophysiology of the disease remains unknown and genetic mutations have been identified in few cases.^6^ Treatment involves intravenous or subcutaneous replacement of immunoglobulins.^2^, ^7^ The use of IVIG has dramatically altered the clinical course of this disease and decreased the burden of recurrent infections, although antibiotics remain essential for managing acute infections.^2^, ^7^

Common variable immunodeficiency patients can be divided into two groups: those who get recurrent infection alone and those who additionally develop non‐infectious complications including multisystemic granulomatous disease, malignancies, and autoimmune diseases.^2^, ^4^ The former group of patients do well with immunoglobulin treatment while the patients who develop non‐infectious complications have poorer health outcomes and a significantly reduced life expectancy.^2^, ^6^ In a study by Resnick et al.,^6^ the mortality rate for CVID patients with one or more of the non‐infectious complications was eleven times higher than for subjects who had infections only.

The incidence of granulomatous disease in CVID patients is approximately 8%–22%.^3^, ^4^ The lungs are the most common organ system affected; however, granulomas are also found frequently in other organs, including the spleen, lymph nodes, liver, gastrointestinal tract, bone marrow, skin, eyes, central nervous system, parotid gland, and kidney.^3^, ^4^ Granulomatous involvement thought to be underdiagnosed because biopsies are performed too infrequently to confirm the diagnosis.^3^

Immunoglobulins are effective in CVID although this treatment is not effective in controlling granulomatous disease.^3^, ^4^ Corticosteroids are usually the first choice to treat granulomatous manifestations, although their effectiveness may be limited.^3^, ^4^ Other treatment options include immunosuppressants such as cyclosporine, hydroxychloroquine, azathioprine, and rituximab.^3^, ^4^ Treatment of the granulomatous disease can be difficult as immune suppression is undesirable in the setting of an underlying immunodeficiency.^4^

Chronic rhinosinusitis (CRS) is a common feature of CVID.^8^, ^9^, ^10^ Oksenhendler found that sinonasal symptoms were one of the most frequent initial symptoms, in 36% of patients with CVID.^9^ Given the prevalence of CRS in CVID, it is important to consider an underlying immune deficiency when treating patients with refractory CRS.^10^ A meta‐analysis by Schwitzguebel et al^11^ that included 1418 individuals with CRS from 13 studies, found that 13% of individuals with recurrent CRS and 23% of patients with difficult‐to‐treat CRS had immunoglobulin deficiencies. While there is literature outlining the prevalence of CRS in CVID, there is no literature to our knowledge reporting granulomatous inflammation affecting the sinonasal mucosa.

Epistaxis from an internal carotid aneurysm is a rare, often fatal event.^12^ Although the exact pathophysiology behind internal carotid aneurysms remains unclear, traumatic (previous sinus surgery and head injury), infectious (mycotic), inflammatory (vasculitis), and congenital origins have been implicated in the development.^13^, ^14^

In 2017, a case report of a young female with an aneurysm in the cavernous segment of the internal carotid artery eroding through the sphenoid sinus was published. She presented with massive epistaxis and was successfully treated with coil embolization. She had suffered from granulomatosis with polyangiitis (GPA), which likely contributed to the erosion of the sphenoid sinus, although they concluded that they were uncertain whether her risk of developing an intracranial aneurysm was any higher than the general population.^12^

Another case has been reported of a 41‐year‐old AIDS patient who presented with severe epistaxis and underwent successful radiological treatment of a ruptured intra‐cavernous internal carotid artery aneurysm.14 Cerebral aneurysms in HIV/AIDS have been classified into two categories: mycotic aneurysms from bacterial or fungal infections, and HIV‐associated aneurysms from a localized vasculitis.^14^

Mycotic aneurysm formation is usually initiated with the seeding of a bacterial or fungal septic embolus within the intracranial arterial vessel wall, most often in the setting of infective endocarditis.^14^ Less commonly it can occur by direct extension to the arterial vessel wall from a contiguous infective source such as the paranasal sinuses.^14^ Local spread of the pathogen leads to vessel wall necrosis and development of an pseudoaneurysm.^14^ HIV‐positive patients, similarly to CVID patients, are likely to be at greater risk of developing mycotic aneurysms because of their immunocompromised state leading to recurrent infection.^14^ On the contrary, HIV‐associated aneurysms are characterized by a vasculitis of the vasa vasorum, a network of small blood vessels that supply the walls of large blood vessels.^14^ The arterial wall is chronically inflamed with fragmentation of the elastic fibers resulting in areas of acute inflammation and transmural necrosis.^14^

The patient in this case report had negative HIV serology, and the cause of his pseudoaneurysm remains unknown. The pseudoaneurysm could have been a result of a localized vasculitis, or secondary to infection and subsequent osteitis affecting the sphenoid and petrous temporal bones.

## 4 CONCLUSION

Granulomatous disease is a manifestation of CVID that may be under‐recognized. Treatment involves immune suppression which is problematic in the setting of a pre‐existing immune defect. Granulomatous inflammation affecting the sinonasal mucosa in patients with CVID has not previously been described in the literature, nor has fatal epistaxis secondary to internal carotid artery pseudoaneurysm.

## CONFLICT OF INTEREST

There is no conflict of interest to declare.

## AUTHOR CONTRIBUTIONS

Lucy Barber involved in writing‐original draft and editing, submitting. Anthony Jordan and Richard Douglas involved in supervision and editing.

## ETHICAL APPROVAL

We confirm that the publication of the case report did not require institutional approval. Written consent for publication was obtained and is available upon request.

## CONSENT

Written informed consent was obtained from the patient's next of kin to publish this report in accordance with the journal's patient consent policy.

## Data Availability

Data sharing is not applicable to this article as no datasets were generated or analyzed during the current study.
